# Exploring the social value of Public Health Institutes: An international scoping survey and expert interviews

**DOI:** 10.3389/fpubh.2022.906286

**Published:** 2022-08-17

**Authors:** Kathryn Ashton, Liz Green, Timo Clemens, Lee Parry-Williams, Mariana Dyakova, Mark A. Bellis

**Affiliations:** ^1^Policy and International Health, World Health Organization Collaborating Centre on Investment for Health and Well-being, Public Health Wales, Cardiff, United Kingdom; ^2^Department of International Health, Faculty of Health, Medicine and Life Sciences, School CAPHRI (Care and Public Health Research Institute), Maastricht, Netherlands

**Keywords:** social value, Social Return on Investment (SROI), Public Health Institutes, wider determinants of health, economic evaluation

## Abstract

**Introduction:**

Making the case for investing in preventative public health by illustrating not only the health impact but the social, economic and environmental value of Public Health Institutes is imperative. This is captured by the concept of Social Value, which when measured, demonstrates the combined intersectoral value of public health. There is currently insufficient research and evidence to show the social value of Public Health Institutes and their work across the life course, population groups and settings, in order to make the case for more investment.

**Methods:**

During July 2021, a quantitative online self-administered questionnaire was conducted across international networks. Semi-structured interviews were also carried out with nine representatives to gain a deeper understanding. A thematic analysis was undertaken on the data collected.

**Results:**

In total, 82.3% (*n* = 14) were aware of the terminology of social value and 58.8% (*n* = 10) were aware of the economic method of Social Return on Investment. However, only two Institutes reported capturing social and community impacts within their economic analysis and only 41.2% (*n* = 7) currently capture or measure the social value of their actions. Interviews and survey responses indicate a lack of resources, skills and buy-in from political powers. Finally, 76.5% (*n* = 12) wanted to do more to understand and measure wider outcomes and impact of their actions. It was noted this can be achieved through enhancing political will, developing a community of best practice and tools.

**Conclusion:**

This research can inform future work to understand how to measure the holistic social value of Public Health Institutes, in order to strengthen institutional capacity and impact, as well as to achieve a more equitable society, and a more sustainable health system and economy, making the case for investing in public health, as we recover from COVID-19.

## Introduction

All nations experience challenges to their population's health and wellbeing which emerge from preventable causes (1). The COVID-19 pandemic has created a global revolution of social, environmental and economic imbalance, exacerbating existing health inequalities and generating new challenges unequally across society. These threats have highlighted the interdependence between population, societal, economic and environmental factors, and the need for greater investment in health and wellbeing (2–4). A key mechanism in investment to help tackle these inequalities, and to the improvement and protection of population health is through the discipline of public health (5). Public health action contributes greatly to disease prevention, health promotion and the prolongation of life amongst populations as a whole. This is achieved by supporting individuals, organizations and populations to tackle preventable disease, mortality and disability using methods of prevention, protection, and promotion (6).

During the last century, Public Health Institutes have materialized to address enduring and emergency public health challenges at national or local levels (7). They have been defined as an organizational unit of a national or regional government health ministry which serves the whole country or region as a source of independent technical public health expertise, coordination of activities, and provides science-based leadership (8). This definition can vary by country, and although Public Health Institutes around the world differ by name, structure, size and scope, their focus on the core public health functions at national and subnational levels is the cross-cutting commonality (5, 7). Epidemics and pandemics such as SARS, Ebola and COVID-19 have highlighted the importance of public health and the role Public Health Institutes play. This has encouraged nations to increasingly establish their own Institutes (7, 9). For example, the Public Health Agency of Canada was created in 2004 following the 2002–2003 SARS outbreak (1). The scope of these Institutes has expanded over the years from health protection due to developments of new concepts such as the wider determinants of health (10) to include more complex and multidisciplinary challenges such as non-communicable disease and health equity (7).

The value of public health systems may seem obvious in the light of progress in public health over recent decades (11, 12). However, in order to justify continued and potential increased investment in these Institutes, it is necessary to explicitly make the case for investing in preventative public health action by collectively illustrating not only their health impact but their social, economic and environmental value. This has been recognized in the “Health in All Policies” approach which acknowledges that health is impacted by, and can affect, cross-cutting policy areas for example, the economy or the environment (13). As we transfer into the recovery phase from the COVID-19 pandemic, it is important to maximize the value of Public Health Institutes, their social responsibility and activities, incorporating social, economic and environmental outcomes into decision-making processes across sectors, promoting them as an investment rather than a cost.

Building evidence on the social and economic value of health and actions to address inequalities has historically been difficult to measure (12, 14). Traditional methods of valuation focus on capturing the financial value of the service being delivered as much as their effectiveness. However, the concept of social value has emerged to complement this and articulate wider benefits of them such as the broader human and societal factors that result from an intervention. Social value can be defined as the quantification of the relative importance that people places on the changes they experience in their lives (15). When measured, social value can demonstrate the combined holistic value of public health, including its triple bottom line: social, economic, and environmental benefit. This goes beyond just capturing the financial return, but also includes the potential benefits to the local and national economy, the individuals affected and their families and communities (16). Capturing the social value of Public Health Institutes can strengthen their national impact, taking forward international, national and local commitments and enabling sustainable and fair policy and action for the benefit of people, communities, the national and local economy, and the planet.

New economic methodologies have emerged to help capture and value the wider societal impact of different interventions, service and policies, such as Social Return on Investment (SROI) (17). SROI is defined as an economic method which measures and captures social, economic and environmental costs and benefits. Unlike traditional economics methodologies, such as cost-effectiveness and cost-benefit analyses, SROI is looking also at non-financial impacts that add real value to people's lives, to communities and society, and to the wider economic and environmental setting. Through stakeholder engagement, the SROI approach defines outcomes, and allows a monetary value to be placed on the non-financial returns on investment by applying proxy values (18). This is particularly relevant for public health policies and interventions, which usually have multiple “soft” and difficult to quantify impacts, such as improving or promoting health, wellbeing and equity of population groups and communities, as well as bringing additional benefits to their living, social, or working environment (19–21).

Exploration is required to understand how the social value and impact of National and Regional Public Health Institutes is, or can be, captured and measured to help promote the wider value of their investment and activities. Previous research has collated existing social value evidence for public health interventions and services (19, 20), and a scoping exercise has been undertaken to gather information from SROI experts on how the concept of social value has been used in the field of public health (19). However, there is currently insufficient evidence which demonstrates the social value of Public Health Institutes and their work across the life course, population groups and settings, in order to make the case for more resources and investment or wider remit of institutes. Previous research has demonstrated the need for more rigorous exploration to inform future institutional development and design (7). Hence, further exploration is required to understand how Public Health Institutes are currently capturing social value, and what tools are being used to do so. The purpose of this paper is to gain a better understanding of how to measure the holistic value of public health services and interventions, in order to strengthen institutional capacity and impact on population health and wellbeing, as well as to achieve a more inclusive society, and a more sustainable health system and economy, as we recover from COVID-19.

## Materials and methods

Due to the explorative nature of this work, an international survey and expert interviews were undertaken to allow for a varied international reach and to enable triangulation of results. The population of interest for survey dissemination were solely National and Regional Public Health Institutes. Other public health organizations and agencies, such as Schools/Faculties of Public Health or third sector bodies, were not targeted. Expert interviews allowed for a deepened exploration of the findings and further clarification of themes identified by the survey.

Based on existing definitions of a Public Health Institute (8), for the purpose of this research, these Institutes are defined as:

“A Public Health Institute is a government agency, or closely networked group of agencies, that provides science-based leadership, expertise, and coordination for a country's or region's public health activities.”

### Survey design and administration

During July 2021, a quantitative online self-administered survey was conducted using Survey Monkey^®^. The survey questionnaire consisted of 45 questions covering background details for their Institute, awareness and experience of economic methods of evaluation, social value and health impact assessment within their Institute, and enablers and barriers to using these methods. The questionnaire was developed with guidance from social value experts from the United Kingdom, and input was provided from the International Association of Public Health Institutes (IANPH). In total, 35 questions were closed, and 10 allowed respondents to input open-ended responses to enable respondents to elaborate on certain questions of interest. The questionnaire was made available in English only. The questionnaire was piloted both internally and externally to Public Health Wales. Written feedback was obtained and integrated into the final version of the questionnaire.

Sampling was based on two non-probability sampling methods; purposive and convenience sampling. In purposive sampling, participants are selected based on a specific characteristic (22). Inclusion criteria in this instance was that all participants had to be formally representing a Public Health Institute with a national or regional remit to provide public health services and interventions to protect, improve and promote public health and are funded by national or regional government.

An email invitation and participant information sheet (which included definitions of key concepts, information on confidentiality and anonymity and what would be done with the results) was circulated *via* established networks. These included IANPHI, the World Health Organization (WHO) networks, the European Public Health Association, and EuroHealthNet. In addition, convenience sampling was used to directly circulate the questionnaire to other known contacts from Public Health Institutes to ask for their participation in the survey. In addition, follow-up reminders were sent halfway through the data collection period.

Approval from an Ethics Committee was not required for this research as per guidance from the NHS Health Research Association ethics decision tool (23). This research posed no potential risk to the individuals participating and all data collected and analyzed was pseudonomysed.

### Semi-structured interviews

Within the questionnaire, respondents were given the opportunity to indicate whether they would be interested in participating in a semi-structured interview. The focus of the interviews was guided by the results of the survey, aiming to add value to the research by gaining a deeper understanding of responses, helping to explore existing practices further, and allowing for triangulation of the information and provided extra insights to help bolster the findings. Consenting individuals were contacted *via* email after survey closure to participate in an interview. Informed consent to participation was collected by the researchers prior to interviewing. Interviews were undertaken *via* virtual video calls or telephone. All interviews were digitally recorded and transcribed. The interviews followed a semi-structured approach, which allowed participants to describe their experiences and expertise at length, but participants were gently guided to discuss areas of particular interest.

### Analysis

Analysis of closed response questions from the survey was undertaken in Microsoft Excel. Opened ended responses from the survey and responses to the interview questions were analyzed thematically (24) by two members of the research team.

## Results

### Study participants

The survey ran from the 7 to 19th July 2021. In total, 37 responses were received. Of these, 12 responses (29.7%) were excluded from analysis because they were incomplete (*n* = 5) or were representing organizations which didn't fit the definition of a Public Health Institute used in this research (*n* = 7). Of the remaining 25 eligible responses, there were representatives from 17 countries. To avoid introducing a country bias where countries or regions had more than one response, answers were merged. In total, 76.5% (*n* = 13) respondents were based in European Institutes, 11.8% (*n* = 2) were based in Oceania, and 11.8% (*n* = 2) in Asia.

In addition, nine interviews were completed throughout September and October 2021. The number of interviews satisfied the needs of this scoping study to enable the presentation of the key emerging themes. Of the interview participants, two noted they had not been aware of the term “social value” prior to being approached for this study.

### Awareness and understanding of the key concepts

When questioned, survey representatives from all responding Institutes indicated that their organization advocates for investment in public health (*n* = 17). Respondents indicated that they do this in a range of ways, for example using existing evidence resources and public health indicators to promote importance and awareness and aligning work with the Sustainable Development Goals and other key policies.

Before being asked to complete the survey, 82.3% (*n* = 14) indicated they were aware of the term social value. Those who indicated no awareness were from Northern Europe. Of the 14, 92.9% (*n* = 13) knew of the definition of the term social value. Overall, 58.8% (*n* = 10) of respondents indicated awareness of the economic methodology of SROI, and of these, 50% (*n* = 5) respondents were aware of their Institute having used SROI to capture the social value of a programme or intervention. In addition, all interview respondents recognized social value aims to capture and measure the impact on the wider determinants of health, not just the economic:

*It combines social, economic, and environmental sort of impacts and benefits and out, outcomes, of different interventions or programmes. (Interview response)*

### Current use of economic evaluation and SROI in national and regional public health institutes

In total, 58.8% of survey respondents (*n* = 10) stated all or some of their programmes and services are informed using economic evaluation. Of these economic evaluations, 70% (*n* = 7) considered physical or mental health impacts, 40% (*n* = 4) considered social and community impacts and 30% (*n* = 3) considered environmental impacts. Furthermore, only 23.5% of responding Institutes (*n* = 4) stated having a dedicated lead for economic evaluation.

Just under half of the Institutes who responded to the survey reported that their Institute currently captures or measures the social value of public health interventions or programmes (41.2%, *n* = 7). Only two Institutes reported using SROI as a methodology to measure this.

In response to current use of economic evidence, four interview participants acknowledged that their Institute uses the best available economic evidence to help inform service development, and four indicated how economic evaluation has been accepted as an important evidence base. However, a strong theme emerged that nuanced the survey results was that this was done mostly on clinical services rather than community-based interventions. Only one interview participant stated there was a dedicated resource to social value in their Institute.

### Perceived benefits and barriers to capturing and measuring social value

A number of benefits and barriers to capturing and measuring social value were reported by both survey respondents and interview participants. Table 1 outlines responses received from survey respondents which were delved into further through the interview process.

**Table 1:** Perceived benefits of and barriers reported by survey respondents to capturing and measuring social value.

**Perceived benefits**	**Perceived barriers**
• Enable organizations to act o*n* the social determinants of health (*n* = 14) • Capturing the social outcomes and impacts (*n* = 14) • Improve service desig*n* and delivery (*n* = 12) • Greater stakeholder engagement (*n* = 12) • Make the case for investing i*n* public health, based o*n* evidence (*n* = 12) • Being accountable to stakeholders (*n* = 9) • Quantifying and monetizing outcomes (showing their financial value) (*n* = 8) • Capturing environmental outcomes (*n* = 5) • Being accountable to funders (*n* = 4)	• Lack of training (*n* = 13) • Lack of resources (*n* = 13) • Lack of awareness (*n* = 10) • Lack of capacity (*n* = 10) • Not a priority at present for the Institute or Government (*n* = 10)

Two interview respondents reiterated the importance of understanding the wider impact of public health initiatives and acknowledged that Public Health Institutes should be validly measuring the wider influences surrounding the potential impacts their programmes and services can have:

*The biggest fraction is around personal and community and, and social value, which combines, obviously benefits to the individual in terms of improving health and mental wellbeing, benefits to the family, to the communities, and… benefits in terms of the, again, increased productivity, reduced, you know, absences… as well as environmental (Interview response)*.

A number of interview respondents (*n* = 3) also reflected on how capturing social value allows an Institute to assess both qualitative date through engagement, but also applying quantitative proxies onto outcomes:

*Provides a way of sort of quantifying things that are not so easily quantified, that are actually of, of high value… a lot of equity related impacts are not always easily sort of quantifiable or able to be monetized, and like, this provides a way of, of doing that (Interview response)*.

The interview respondents reiterated many of the barriers to capturing and measuring social value, with the main theme identified as a lack of common understanding or clear definition of social value internationally:

*I think we should be aware of the—the same concepts with different cultures could be also understood and perceived as differently, even if we read the same words (Interview response)*.

Six Institutes also reported a lack of awareness, capacity and professional knowledge about how to measure social value, particularly using SROI as a relatively new methodology. This was not only reflected to be at the Institute level, but also with regards to policy and decision makers:

*Limited resources and also we are lacking in terms of like the financial side or, you know, economic migration or something like that we need. We need also the professional knowledge within our institute to do that (Interview response)*.

### What could be done to help capture and measure social value?

In total, 76.5% (*n* = 12) of survey respondents stated they would like to do more to understand and measure the wider outcomes and impacts that public health programmes have. Of these, all acknowledged that targeted resources to increase skills and knowledge are needed to progress. In addition, 92% (*n* = 11) indicated that examples of good practice and case studies would be beneficial, 75% (*n* = 9) suggested specialist training, 50% (*n* = 6) reported more needs to be done to improve awareness and 12.5% (*n* = 4) stated that a change in culture within Public Health Institutes was required.

The themes identified through the survey were replicated by the interview participants. Interviewees suggested the need for case studies, tool and templates to help build a community of practice of social value in the field of public health, which would in turn present opportunities for political buy-in and additional resources and also demonstrating the validity of methodologies to capture:

*And to, you know, is, in all these things, particularly in small countries, I think you need a larger country to say, you know, we think this is useful, this is robust, and it can develop into best practice and it's more likely to be adopted (Interview response)*.

*You need to get to critical mass around a topic, you know?... start with very simple things like having a conference, a large international conference of best practice in the area. You then look at, maybe, you know, undertaking some sort of pilot work in the area (Interview response)*.

Improving awareness of economic methodologies such as SROI was a strong theme identified through the interview responses. It was noted by several interviewees that awareness raising needs to be targeted, promoted and disseminated to audiences to have the most impact. Collaboration internationally and with other sectors such as academia and the third sector were also reported to be beneficial to raising awareness of SROI:

*SROI is something I'm familiar with, but I suppose what would be useful is understanding what is different about the method from other ways of evaluating impact so that we could understand what would be the added benefit of using it (Interview response)*.

## Discussion

There is a growing body of literature to support Public Health Institutes being recognized for their capacity and ability to respond to public health emergencies, which has been enhanced and accelerated by COVID-19 (7, 11, 12, 25–27). Overall, this study has highlighted the need and desire for further developments in how Public Health Institutes at the regional and national level capture, measure, and demonstrate their social value and impact. The sample size achieved in the study was deemed acceptable for the scoping nature of this innovative work to gain an initial understanding of the awareness of social value within these Institutes. The consistent themes highlighted across the questions indicate the responding sample show consensus in their thoughts and experiences.

The research highlighted a good overall awareness of the concept of social value and economic methodologies were reported. However, there was a lack of awareness reported on how these concepts and methods could be utilized in practice. Under 50% of responding Institutes stated that they currently measure or capture social value in some respect. But, it is important to note the differing definitions of social value used within Institutions and that the focus of current economic evaluation tends to be driven toward capturing the value of clinical interventions through traditional health economic methodologies such as cost-benefit analysis and cost-effectiveness analysis, rather than community or health promotion services and programmes. This aligns with previous research which indicates that the value of non-clinical health and wellbeing initiatives with a focus on primary prevention are tricky to measure (12).

Both the survey and the interviews allowed this study to scope the understanding of the benefits and barriers to capturing and measuring social value. There was a consensus that capturing social value can improve service delivery and design, help to make the case for investment in public health and also improve stakeholder engagement and accountability to those stakeholders. However, barriers were noted as a lack of capacity, skills, awareness and resources in Institutes to help promote social value as a way of working. This corresponds with previous research on traditional methods of capturing value in public health where a general awareness of methods was reported, but report a lack of skills, resources or capacity to apply them (12).

The results from this study identify the need for the development of a ‘community of practice' to enable Institutes to have the template to pursue capturing and the measuring their own social value. Results show that there is a drive to do this, but support and guidance is needed. This finding is supported by previous research which also highlight the need to dissolve the gap between health economic specialists and public health practitioners on the ground (12). This links to also being able to communicate the social return of investment to key stakeholders and politicians to ensure results are interpreted in the appropriate and desired manner. Key themes identified were the development of case studies, training, templates and tools to help support Institutes, and collaboration with academia and specialist organizations was key to success. Benefits of networks such as the International Association of National Public Health Institutes (IANPHI) (28) and Social Value International (29) could be used as a stepping-stone to help build a “community of practice.”

Within the current global climate, there is growing momentum within international nations to shift the focus from traditional economics to building an “Economy of Wellbeing;” a type of recovery that places health and wellbeing, social, economic, and environmental co-benefits of investment as vital to rebuilding economics and societies in a sustainable and inclusive way (30). This may mean thinking about health differently as things that may look simple from a narrow historical health perspective may be far more complex under a social value and SROI approach, by placing emphasis on those wider indirect outcomes. Public Health Institutes are key actors within this incorporating a holistic viewpoint, and this study can act as the platform to help build knowledge and expertise to progress capturing the co-benefits of investing in this area. This has the potential to encourage decision and policy-makers to see health and wellbeing not as a cost, but as “an investment that is the foundation of productive, resilient, and stable economies” (31), linking in with the “Health in All Policies” approach. Like social value, promoting and building an “Economy of Wellbeing” places people and their wellbeing at the center, embedding a social value approach and tools, which considers all health, economic, social and environmental outcomes of services, and ensures both the economic and the SROI is captured and considered in order to create a more equitable, healthier and more sustainable economy and livelihoods for all.

Although this paper has taken the first steps to explore how Public Health Institutes are capturing the social value of their activities, there are limitations of this research which are important to note. The pattern of country response may be a result of the survey only being made available in English, or due to the reach of the networks through which the survey was promoted. According to IANPHI, there are 110 National Public Health Institutes, which results in a survey response rate of 15.4%. This a respectable response rate given the current climate of COVID-19 and satisfies the exploratory nature of this research. In comparison, a membership survey undertaken by the IANPHI Secretariat pre-COVID-19 gained a response rate of 39% (*n* = 44) (25). Yet, it is important to acknowledge that the achieved sample size did not allow for cross-continent comparison or comparison of results between high-income countries and low-income countries, to enable analysis to indicate where knowledge in this field of work is being developed. However, the use of and interplay between both survey and interview methodology confirmed findings and provided extra insights.

Future research could potentially reach out to additional Institutes to help bolster the findings and allow for analysis across different regions. To help build on this initial investigative exercise, there is scope to understand how different actors can help support Public Health Institutes to capture their social value. For example, additional scoping research with Schools of Public Health in academic institutions. Future research could also explore the practical application of social value methods in Public Health Institutes, such as use of SROI methodologies as the primary tool for capturing and articulating the social value of interventions which are promoted by Public Health Institutes. Although there is existing research which collates social value evidence relevant to public health (19, 20), there is a need to progress academic evidence of cases studies in the specialty of public health (7).

## Conclusion

This scoping work contributes to the growing evidence base that demonstrates the use of social value methodologies in the field of public health and investment in health and wellbeing (19, 20, 32). By engaging with international Public Health Institutes, the results from the survey and interviews can be used as a starting point to help build a ‘community of practice' to equip Institutes with the knowledge, skills and expertise to capture and measure social value to capture the Social Value of their services and interventions, and to maximize their (social) return on investment and impact toward a more inclusive and sustainable societies and economies.

## Data availability statement

The raw data supporting the conclusions of this article will be made available by the authors, without undue reservation.

## Author contributions

KA and LG developed and designed the research. KA and LP-W conducted the interviews. KA analyzed the data and drafted the manuscript. All authors edited and approved the final manuscript.

## Funding

This research was funded internally by Public Health Wales.

## Conflict of interest

The authors declare that the research was conducted in the absence of any commercial or financial relationships that could be construed as a potential conflict of interest.

## Publisher's note

All claims expressed in this article are solely those of the authors and do not necessarily represent those of their affiliated organizations, or those of the publisher, the editors and the reviewers. Any product that may be evaluated in this article, or claim that may be made by its manufacturer, is not guaranteed or endorsed by the publisher.
